# Supplementary figures and images for: CAPYBARA: A generalizable framework for predicting serological measurements across human cohorts
Source: PLoS Comput Biol. 2026 Mar 30;22(3):e1014129. doi: 10.1371/journal.pcbi.1014129 (PMC13046277; doi:10.1371/journal.pcbi.1014129)

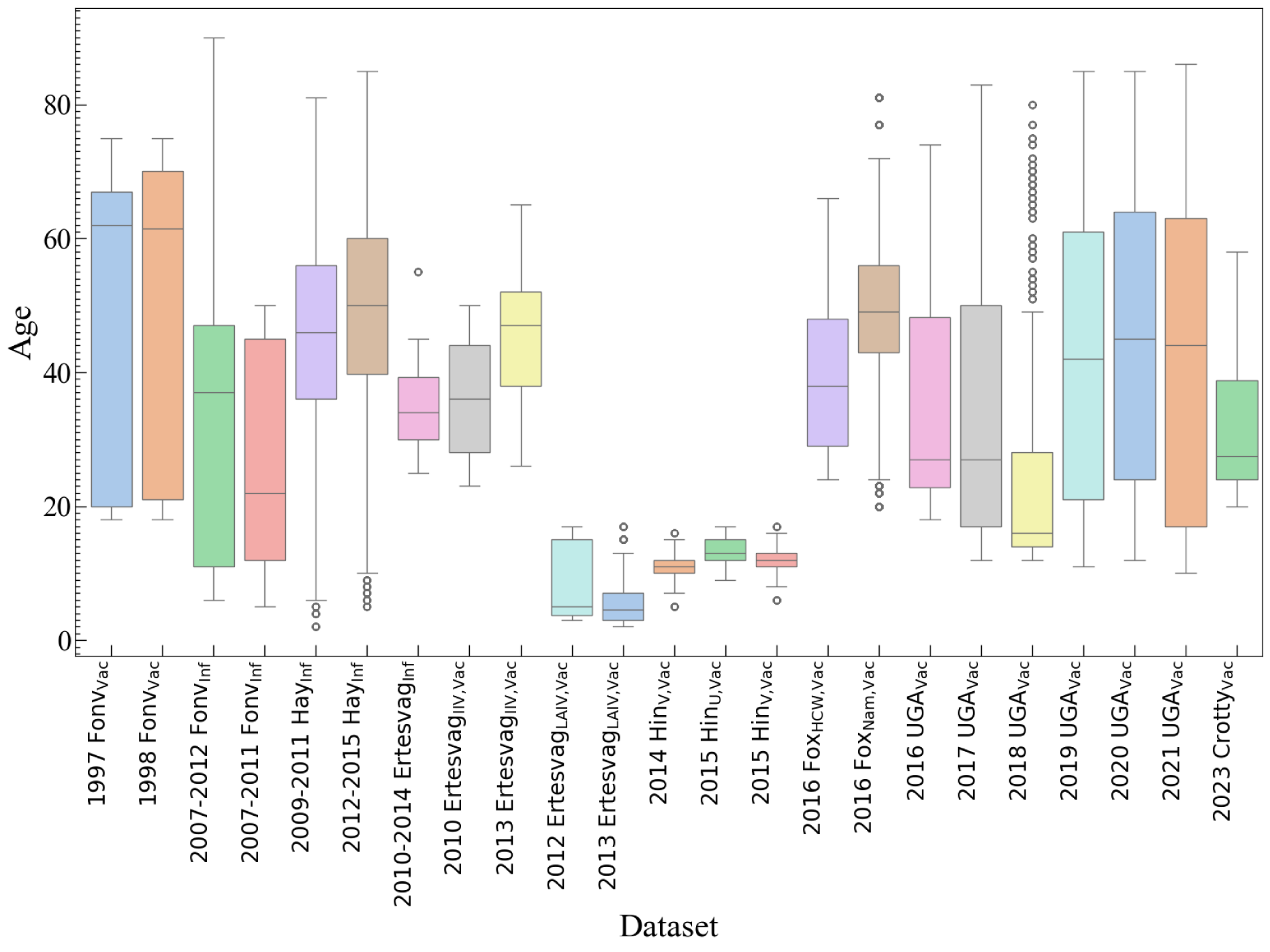

Supplement: S1 Fig — Datasets are ordered chronologically and by study group. (TIF) [file pcbi.1014129.s001.tif]

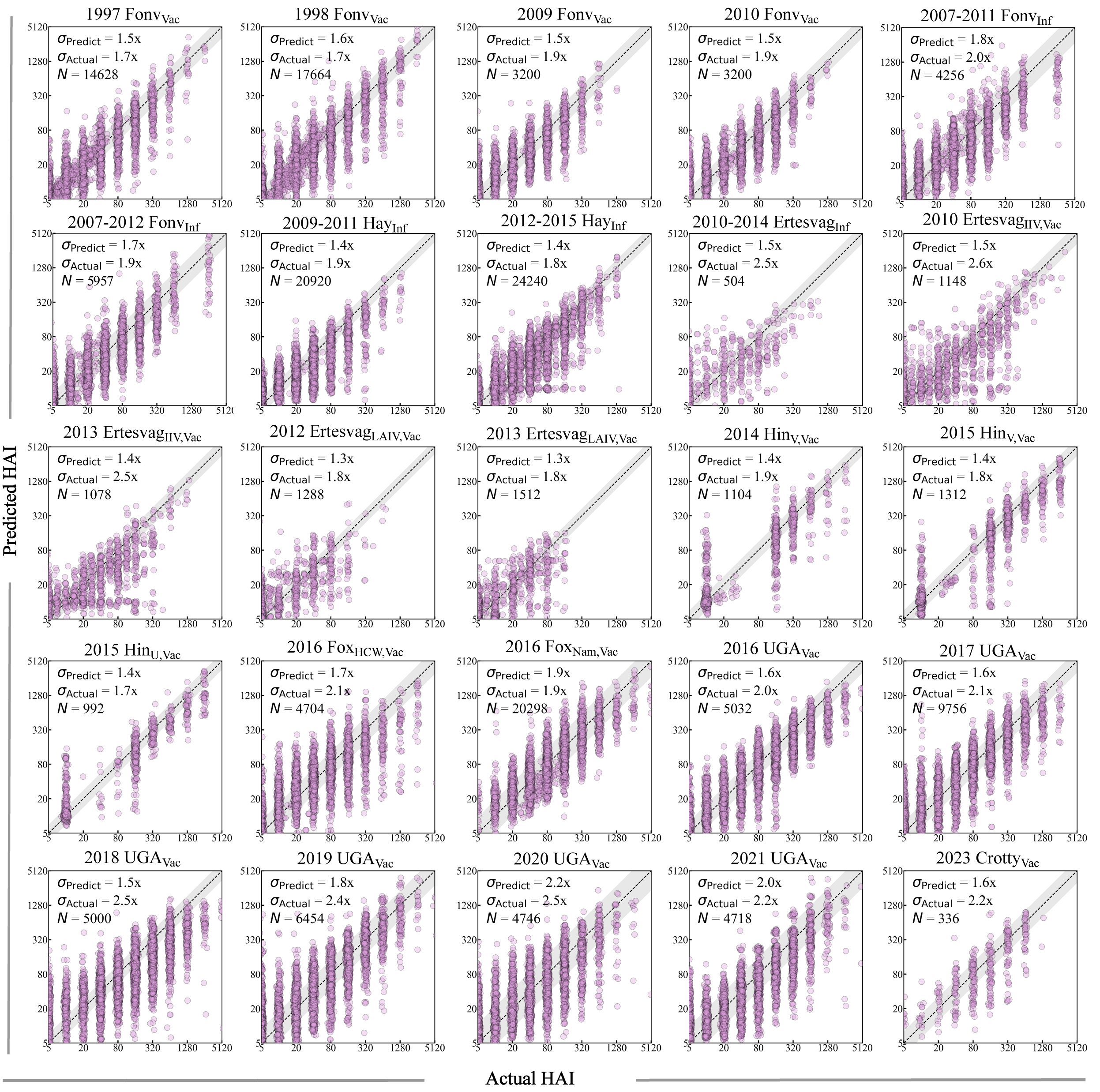

Supplement: S2 Fig — Every other study in Table 2 is used to predict HAI titers for all variants in the study-of-interest (shown by the plot label). (TIF) [file pcbi.1014129.s002.tif]

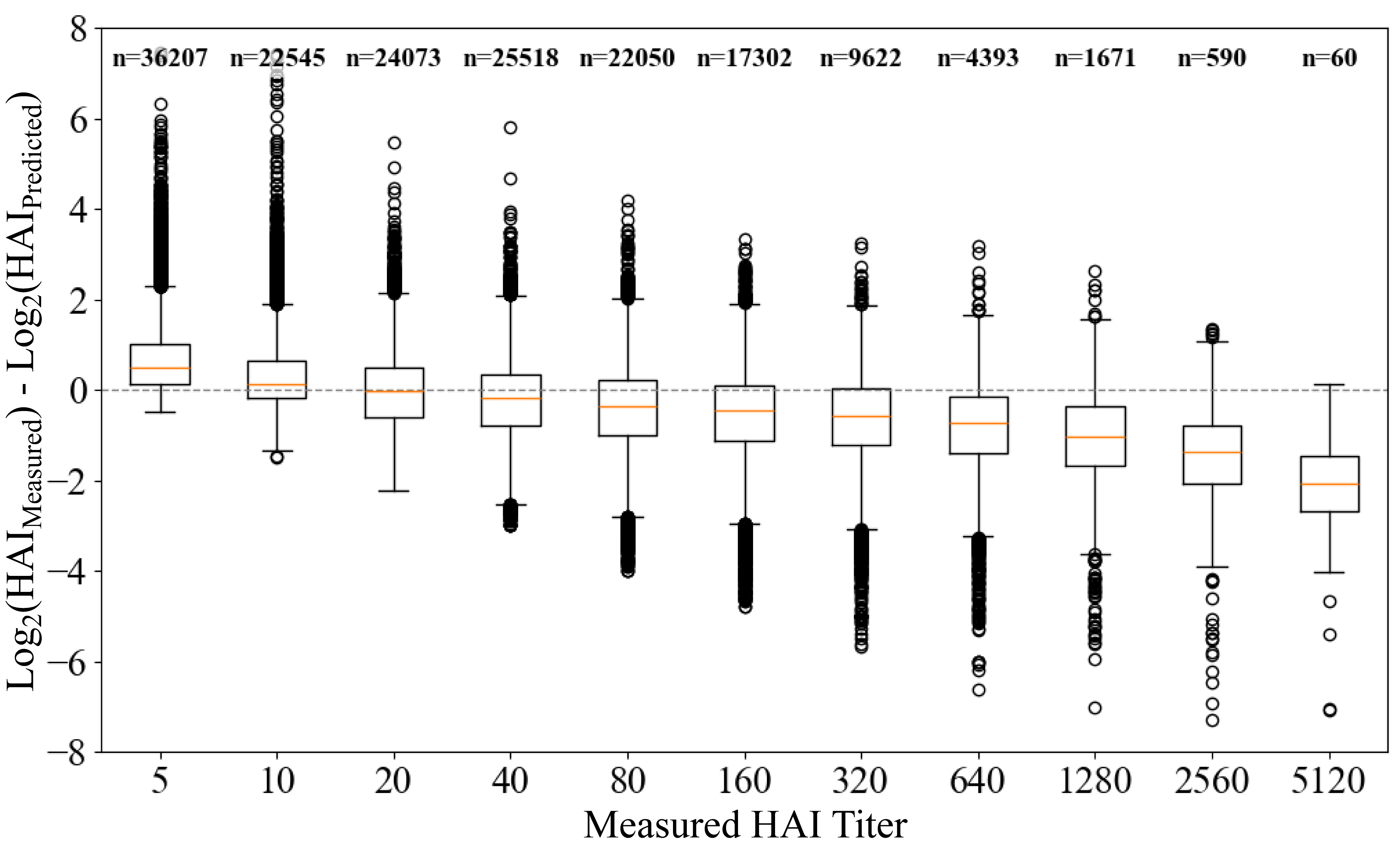

Supplement: S3 Fig — Boxplots show median and interquartile range of prediction residual across all datasets, with sample counts annotated above each bin. (TIF) [file pcbi.1014129.s003.tif]

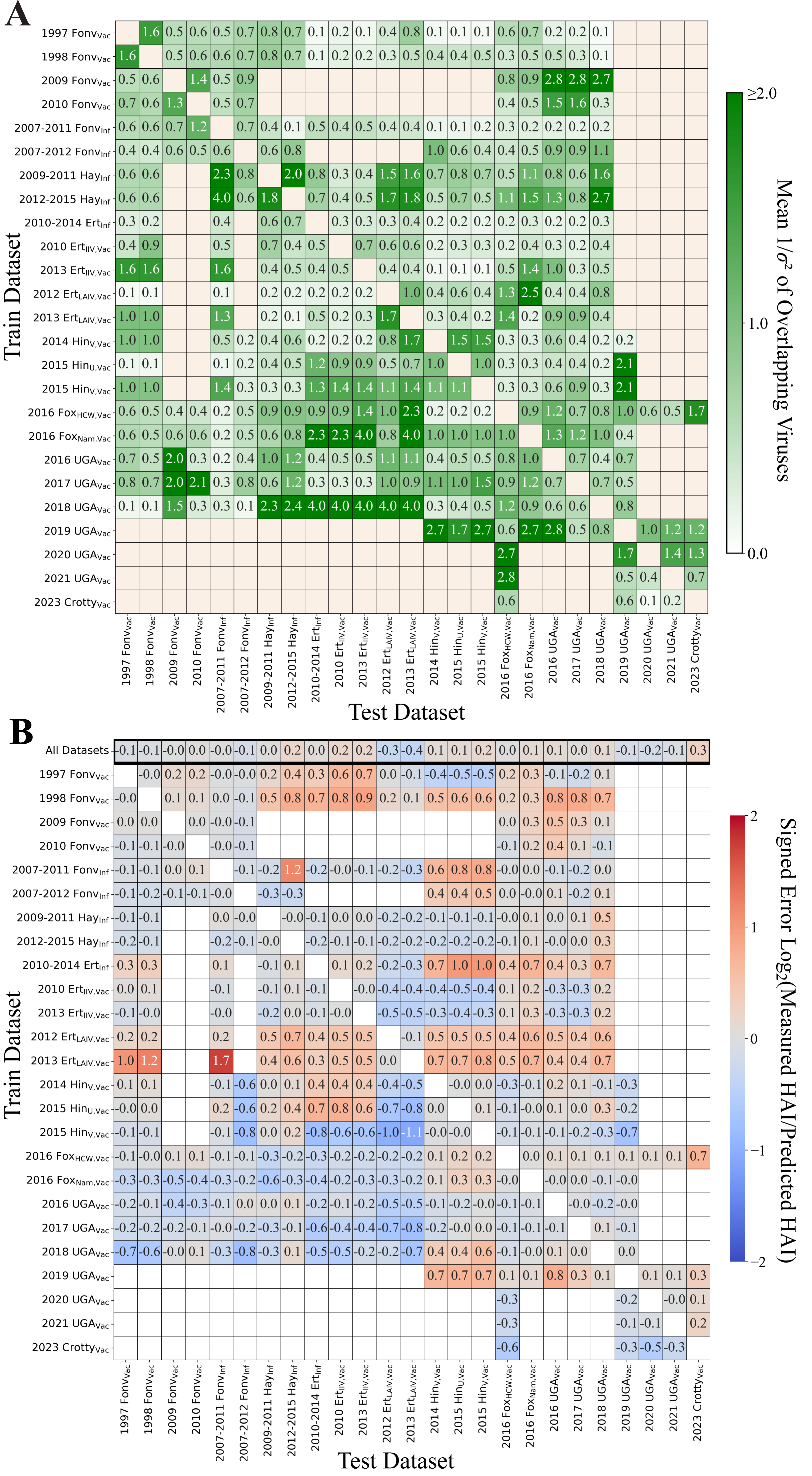

Supplement: S4 Fig — (A) The estimated prediction accuracy for each pair of studies, computed as the mean (1/σPredicted)2 over all overlapping variants. Larger values indicate that the training dataset will be weighted more heavily in combined-study predictions. (B) Signed prediction error on log titers, log2(measured HAI/predicted HAI) for all variants in each pair of studies. Red indicates that measured titers were larger than predicted titers on average. Training is either done using all studies (top row) or using a single study (all other rows). (TIF) [file pcbi.1014129.s004.tif]

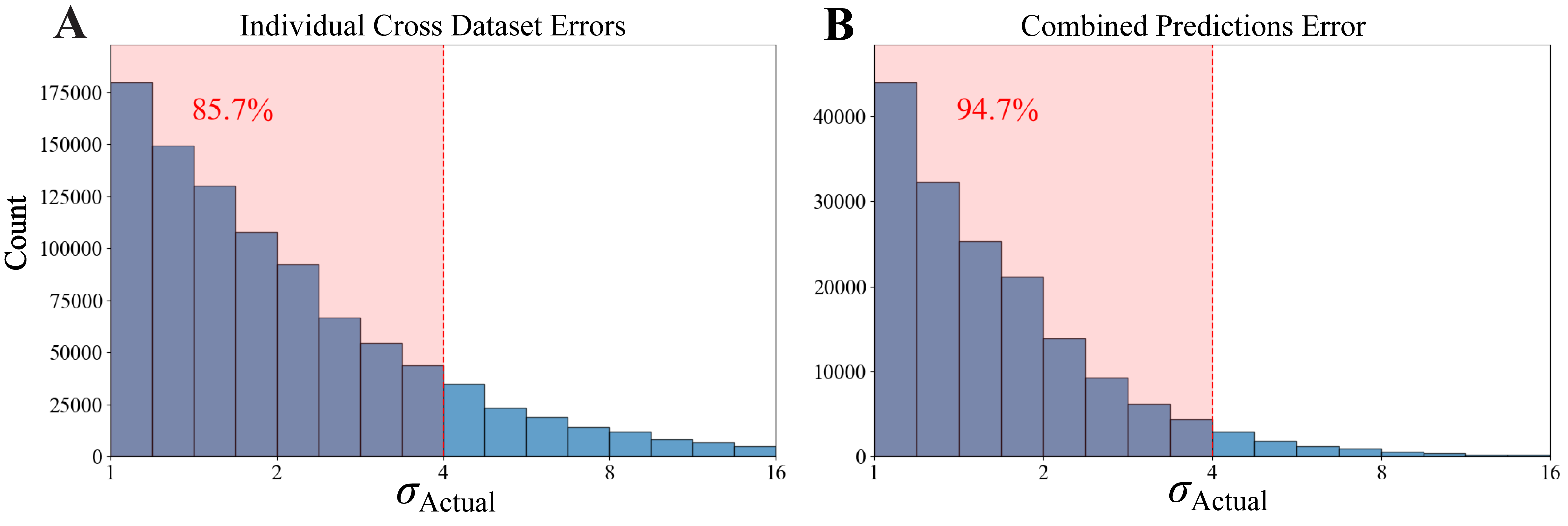

Supplement: S5 Fig — Fold-error (σActual) of predictions for every subject and virus using (A) each dataset to make a separate prediction and (B) all datasets to make combined predictions. Red shading marks the region of ≤4x error, and the annotated percentages indicate the fraction of predictions that fall within this threshold. (TIF) [file pcbi.1014129.s005.tif]

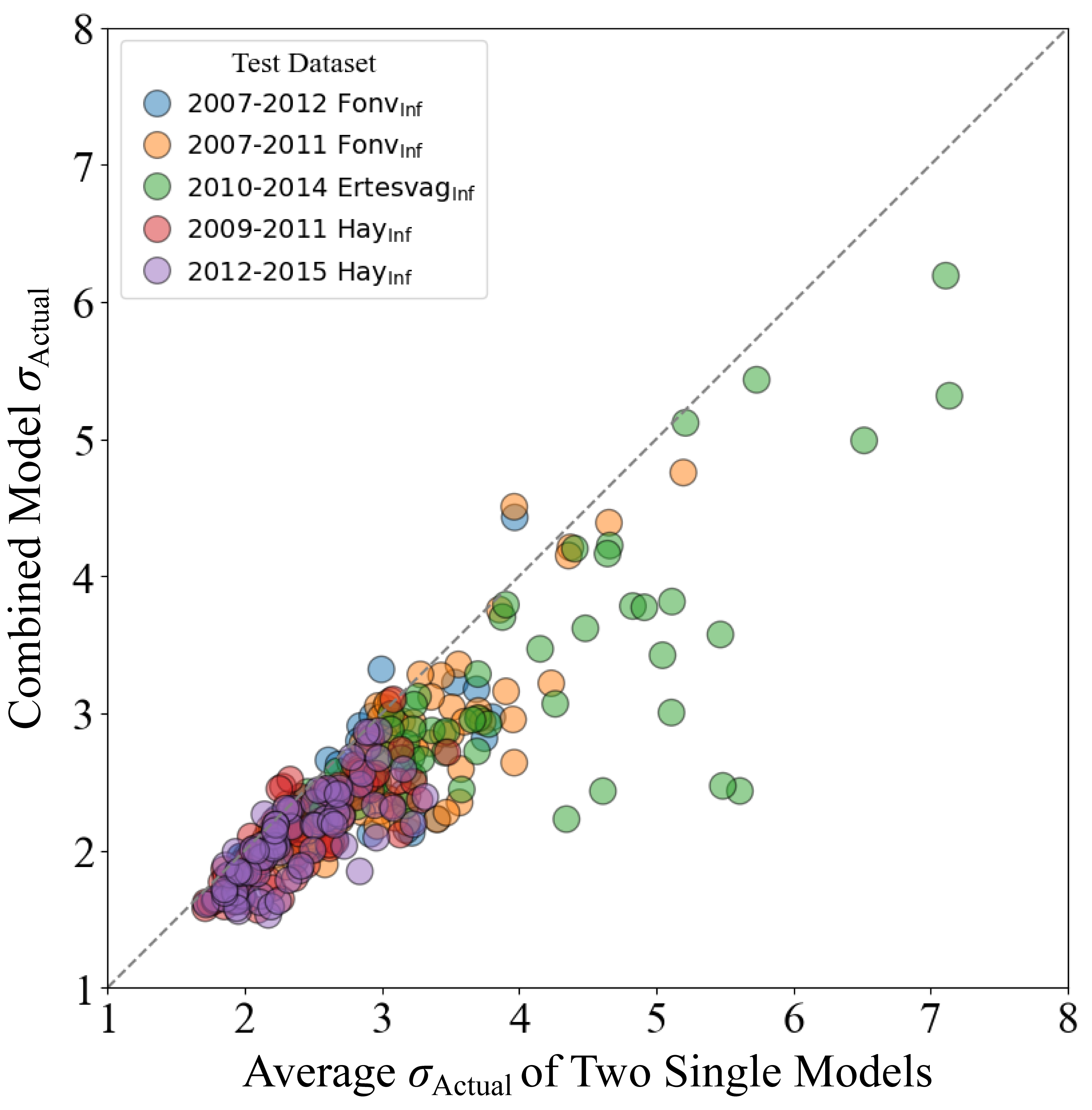

Supplement: S6 Fig — Prediction errors (σActual) for all viruses in all infection studies were computed using two other datasets for training. These two datasets either independently predicted each virus, and their resulting predictions were averaged [x-axis] or CAPYBARA was used to combine these predictions by more heavily weighing the dataset that was more similar to the target infection study [y-axis]. Points below the diagonal indicate improved performance with the combined model. (TIF) [file pcbi.1014129.s006.tif]

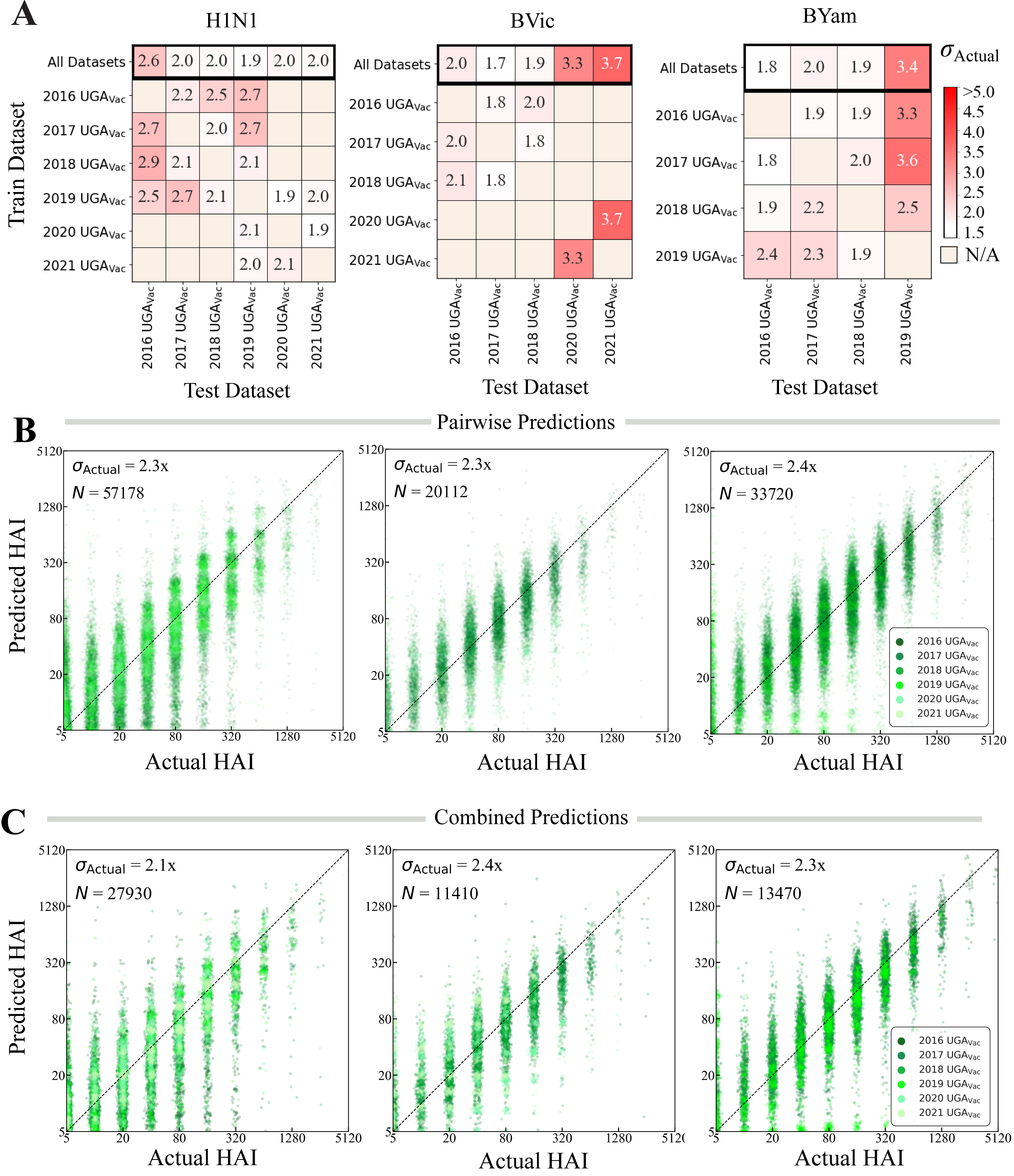

Supplement: S7 Fig — (A) Heatmap of the predicted vs measured RMSE (σActual) across all subjects and overlapping variants for H1N1 (left column), B Victoria (middle column), and B Yamagata (right column). Within each heatmap, training is either done using all studies (top row) or using a single study (all other rows). (B-C) All predicted vs measured HAIs when training on (B) a single study or (C) all other studies. The number N of predictions is larger for pairwise predictions since the same serum-virus pair is predicted multiple times using different training datasets. The diagonal line y = x represents perfect predictions. (TIF) [file pcbi.1014129.s007.tif]

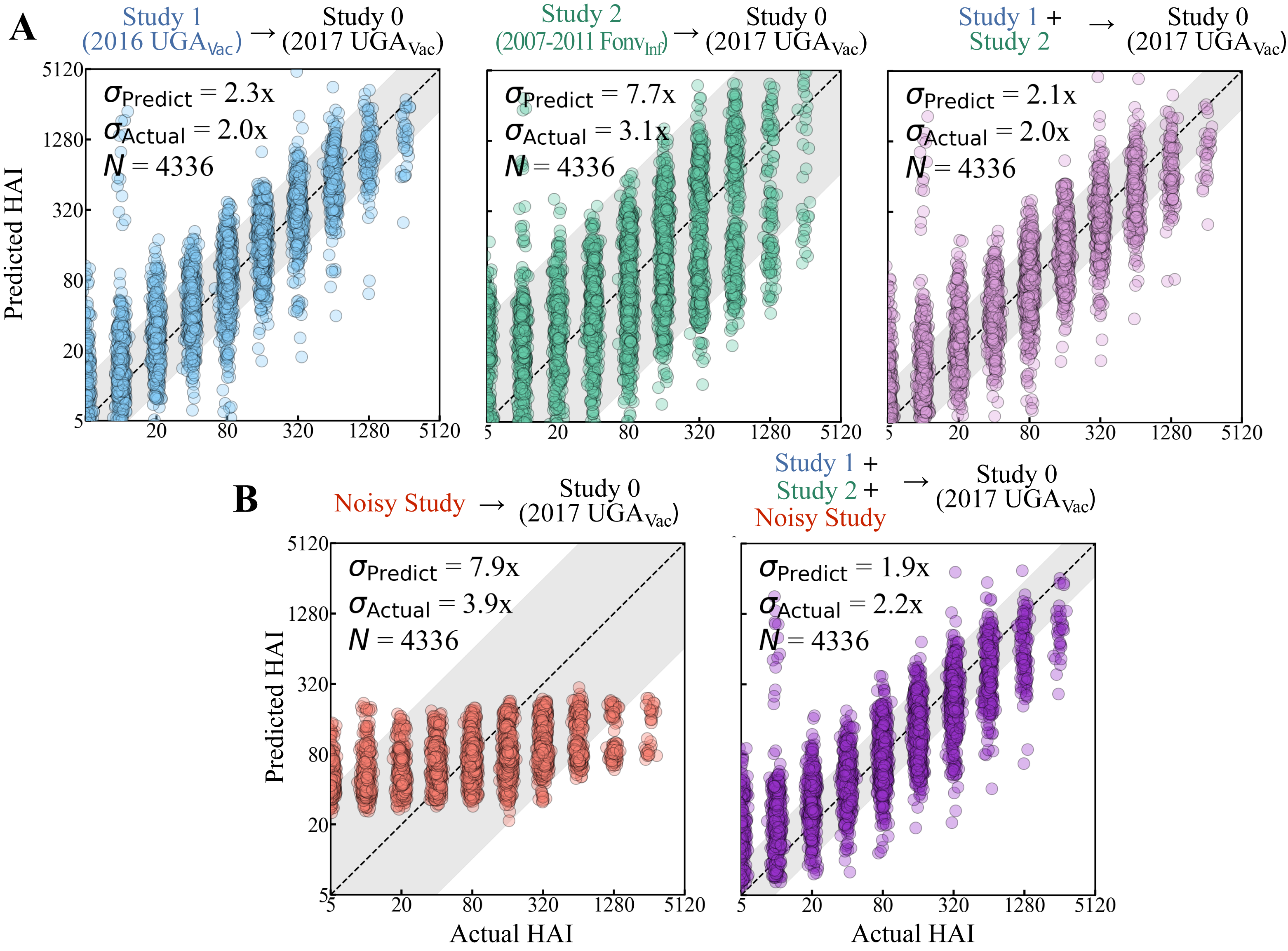

Supplement: S8 Fig — (A) Example predictions from Fig 2A using an individual dataset (left and middle columns) or the combination of both datasets (right column) to predict titers in 2017 UGAVac. Labels above each plot identify the training → testing dataset. (B) A study with random data predicted this same testing dataset either individually (left) or in addition with the other two training datasets from Panel A (right). (TIF) [file pcbi.1014129.s008.tif]

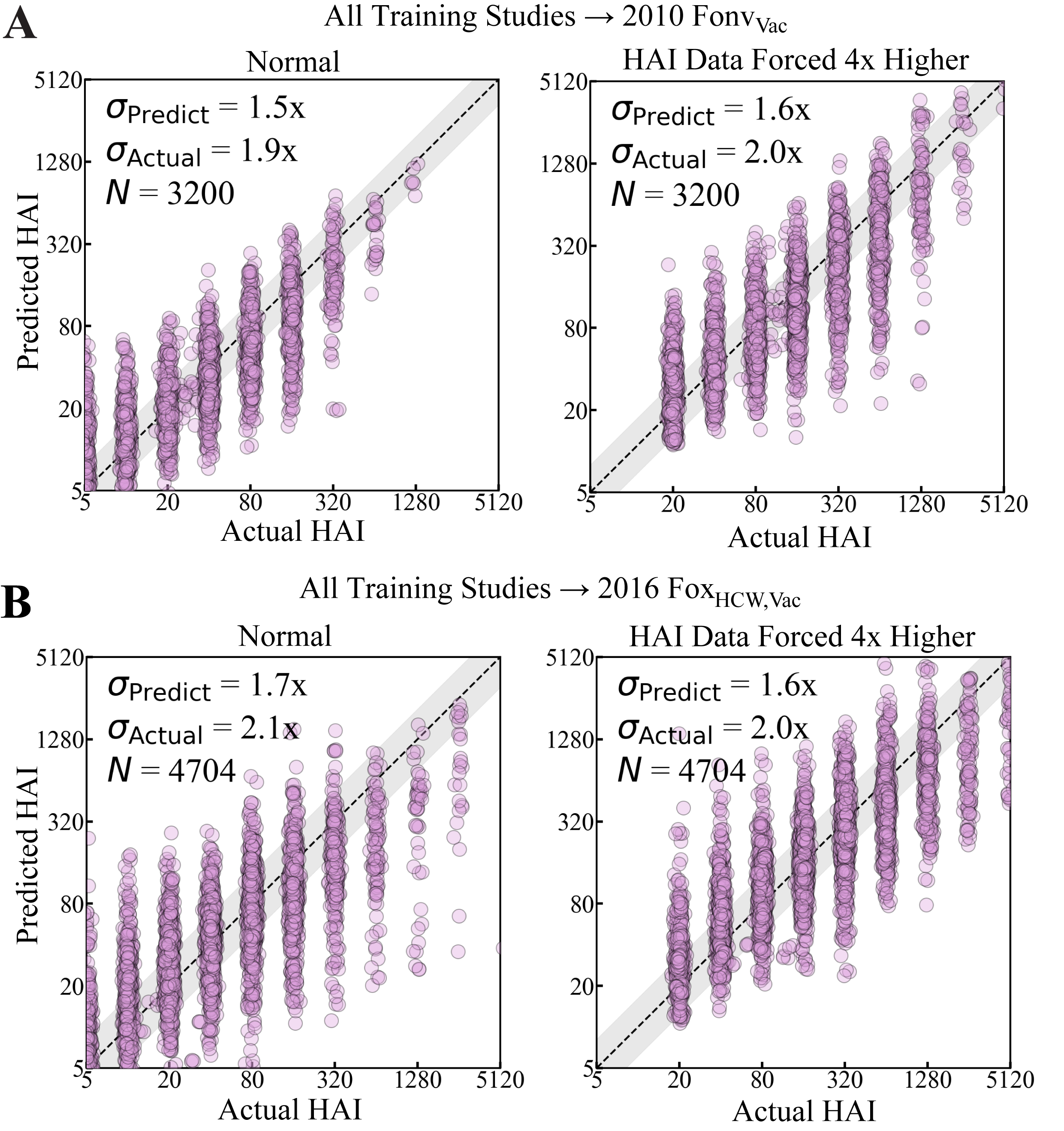

Supplement: S9 Fig — Predicted versus measured HAI titers for two representative vaccine studies based on all other studies. Titers are predicted in (A) 2010 FonvVac and (B) 2016 FoxHCW,Vac using the original data (left) or after multiplying all titers in that single study by 4x (right) to demonstrate the effects of one study having systematically higher titers. (TIF) [file pcbi.1014129.s009.tif]

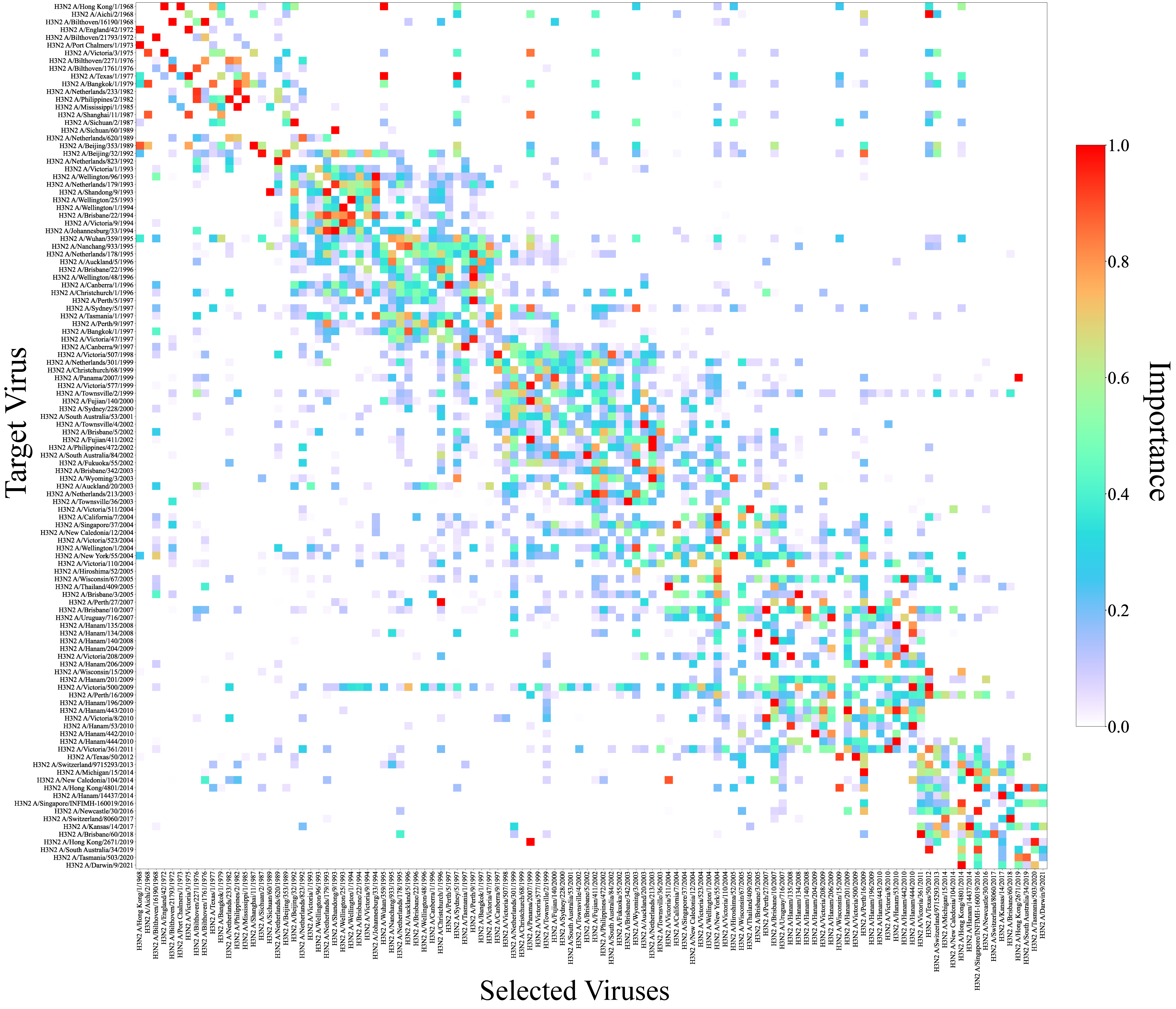

Supplement: S10 Fig — The importance of each virus feature (column) when predicting a target virus (V0, row). Feature importance is quantified within a single study. Only viruses with feature importance≥0.1 shown, as these viruses are subsequently used in ridge regression when predicting the target virus. Any virus not picked is shown in white. (TIF) [file pcbi.1014129.s010.tif]

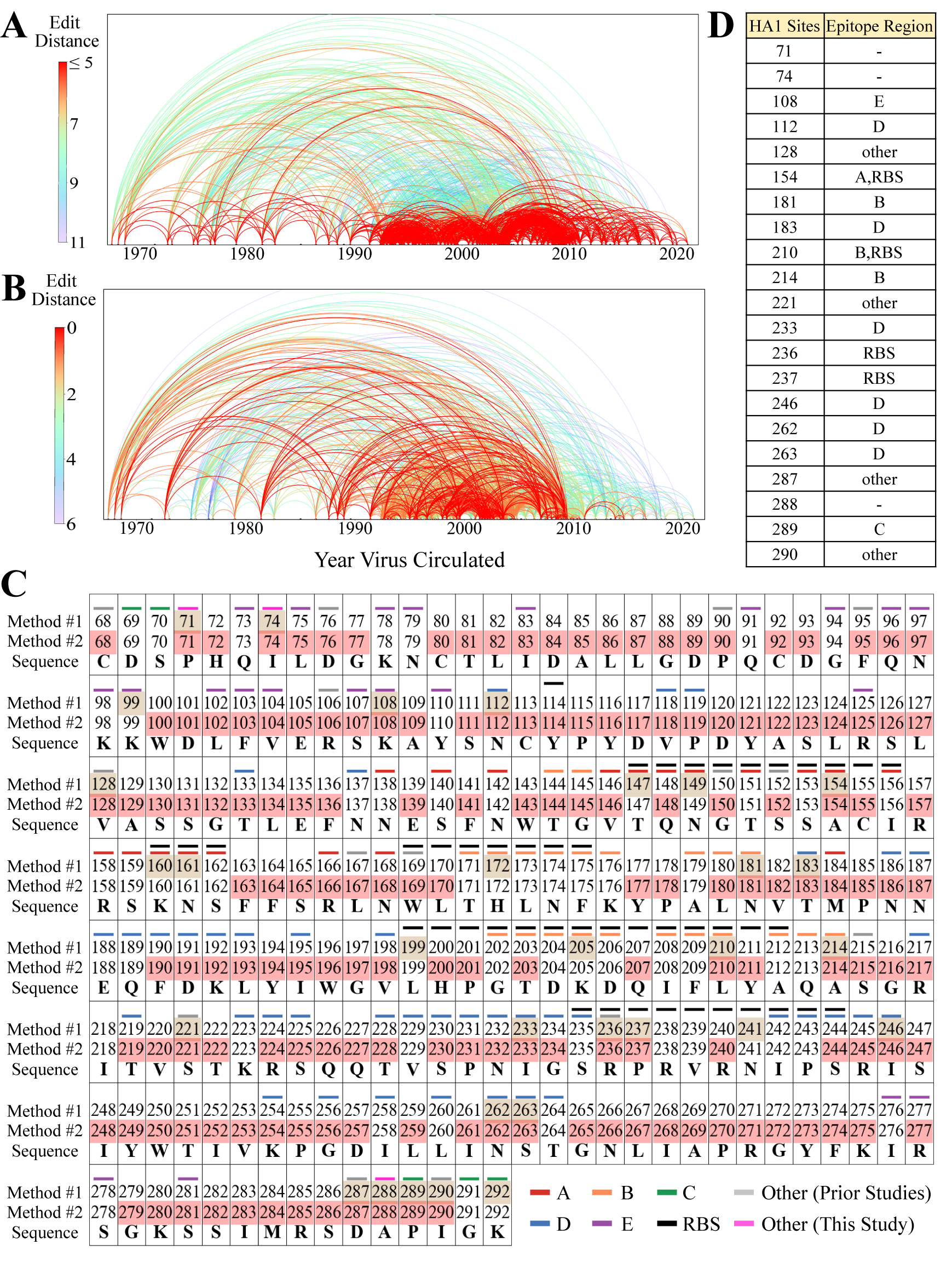

Supplement: S11 Fig — (A-B) Arc plots connecting each virus to its predictive partners. (A) Arcs colored by raw sequence distance computed from the selected 31 positions. (B) Arcs colored by the sequence distance computed from the 167 positions selected from virus pairs 10+ years apart and with high importance (≥0.7) in 80% of pairs. (C) Example sequence H3N2 A/Perth/16/2009 with canonical epitopes denoted by different colored lines above each position, and key residues found from two methods in this study highlighting the corresponding position numbers. (D) List of the 31 HA1 amino acids leading to the minimum Frobenius norm, annotated by which H3N2 epitope they fall into. Amino acid positions include the 16 amino-acid signal peptide. (TIF) [file pcbi.1014129.s011.tif]

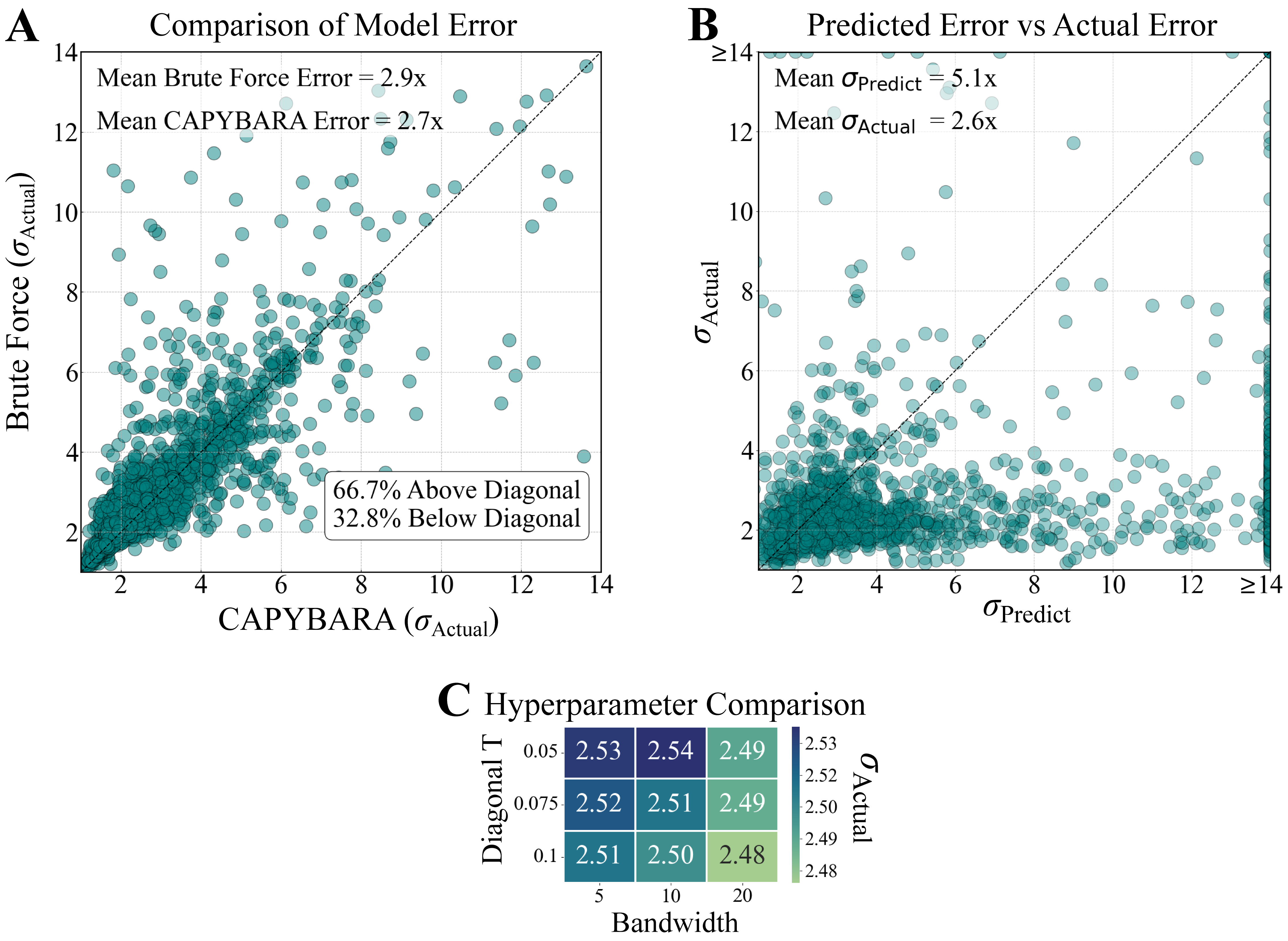

Supplement: S12 Fig — (A) Comparison of fold-error for pairwise models generated by brute-force selection (running ridge regression on five randomly selected viruses, repeating 50 times to find the best five viruses) versus CAPYBARA (runs RFM a single time to identify the most predictive features and then ridge regression). Each point represents an overlapping virus between each dataset pair. More points lie above the diagonal and the average error is slightly smaller along the x-axis, with both traits indicating better performance with CAPYBARA. (B) Predicted versus actual error across all datasets using CAPYBARA, with each point representing all measurements for one virus in one study. We expect the predicted error to represent an upper bound, worst case error (σActual≲σPredict), which is satisfied in the vast majority of cases. (C) Heatmap of mean σActual across all dataset pairs for different hyperparameter settings for the diagonal threshold and bandwidth in RFM, showing nearly comparable prediction accuracy across all parameter choices. (TIF) [file pcbi.1014129.s012.tif]
