# Supplementary material for: CAPYBARA: A generalizable framework for predicting serological measurements across human cohorts
Source: PLoS Comput Biol. 2026 Mar 30;22(3):e1014129. doi: 10.1371/journal.pcbi.1014129 (PMC13046277; doi:10.1371/journal.pcbi.1014129)
Supplement: Attachment — Submitted filename: PLOS_CB_Review___CAPYBARA.pdf [file pcbi.1014129.s014.pdf]

Review of "CAPYBARA: A Generalizable Framework for Predicting Serological Measurements Across Human Cohorts"

August 2025

This paper presents a method for out of sample prediction of serological data, with the ability to combine multiple datasets and preferentially weight studies and factors with more predictive information. This is an important problem in the field as it is highly challenging to reach very large sample sizes in a single human cohort due to practical limitations, so developing methods to incorporate data from and work across multiple cohorts is highly valuable in computational immunology. The code for this work is publically available and well documents, and the manuscript describes the approach somewhat clearly and tests multiple use cases. I have outlined my comments below, but overall I found this to be a nice manuscript.

Major Comments

- I feel it would be very helpful to include a table/graphic/figure explaining the precise definitions of σ_{internal},σ_{external},$\sigma_{\text{predicted}}$, and σ_{actual}. They are defined in the text, but I think this would help with readability. Especially as the relatively low error of the predictions is central to the story of the paper.
- It is a known issue with HAI assays that variability in the assay can come from many factors including site or technician. I wonder if the error estimates generated by this method could be used to develop weights or adjustments between cohorts that could allow them to be combined for other analyses. It would be interesting to see some sort of systematic look at the magnitude and sign of the residuals for particular prediction tasks.
- Tying into my above comment about understanding these errors, by eye it seems the approach has the largest challenges with the highest and lowest titer values. However it may be the case that it is an optical illusion due to all of the errors being in the same direction. Of course, necessarily, error in predicting undetectable titers must be an overestimate. I would

like to see a figure characterizing the error by titer value. It's not an issue in my eye if this is in fact the case, we know HAI limits of detection make these problems challenging, but it would be good to show.

- Is it necessary with this method to impute the missing HAI measurements and predict them/use them for prediction? If it is necessary to do the ridge regressions or feature selection that is okay, but it feels important to acknowledge this.

Minor Comments

- Extra word: "This determines how the error relationship when predicting from Sj to S0 (Fig 1C)..."
- "Prediction accuracy can only decrease as more datasets are included". Increased?
