# Supplementary material for: CAPYBARA: A generalizable framework for predicting serological measurements across human cohorts
Source: PLoS Comput Biol. 2026 Mar 30;22(3):e1014129. doi: 10.1371/journal.pcbi.1014129 (PMC13046277; doi:10.1371/journal.pcbi.1014129)
Supplement: Attachment — Submitted filename: PLOS_CB_Review___CAPYBARA_2.pdf [file pcbi.1014129.s016.pdf]

Review of "CAPYBARA: A Generalizable Framework for Predicting Serological Measurements Across Human Cohorts"

January 2026

I would like to congratulate the authors on responding to the breadth of feedback (with which I largely agreed) in a deep and careful way. After reviewing the changes I feel I can say the additional figures and analyses improved my understanding of the methods and findings. Below are a small handful of clarifications I found while reviewing the additions to the manuscript which the authors may be interested to address but do not materially affect the quality of the paper. I am excited to see this work published.

Comments

- Missing comma in author summary: "Although these studies were conducted over two decades**,** used different study designs,..."
- Last line of author summary I think you need: "thereby leverage" to become "thereby leveraging" or "to thereby leverage"
- Speed is mentioned as a factor which the authors optimized over when designing the CAPYBARA architecture. I would be interested to see run times reported for this approach vs. others tried. It doesn't even need to be figure, just a rough understanding of how long these estimates take to generate
