# Supplementary material for: CAPYBARA: A generalizable framework for predicting serological measurements across human cohorts
Source: PLoS Comput Biol. 2026 Mar 30;22(3):e1014129. doi: 10.1371/journal.pcbi.1014129 (PMC13046277; doi:10.1371/journal.pcbi.1014129)
Supplement: Attachment — Submitted filename: Response to Reviewers.docx [file pcbi.1014129.s017.docx]

We thank the Reviewers for their feedback, which has strengthened the paper and made it more accessible. We address each Reviewer’s comments point-by-point below, and line numbers are provided that match the version of the manuscript with tracked changes.

Reviewer #1

This manuscript develops an algorithm to predict HAI titers to a large number of variants using serological measures of only a few variants, by exploiting cross-reactivity information from historical data sets. A significant limitation is that the variant of interest must already exist in the historical data set, and the need to test at least some variants in the current study — unlike ab initio models based on viral sequence like in Loes et al (Ref #6). The CAPYBARA algorithm could potentially be useful in reducing cost and labor, and for understanding the landscape of cross-reactivity, but the latter is not fully explored.

Major Comments:
1) The algorithm is rather ad-hoc in nature, combining approaches from ML (RFM), frequentist statistics (ridge regression), and Bayesian statistics (study weighting). Depending on the reader’s preference for methodological consistency, this is either a trivial or serious issue.

*Author response*: This is an accurate summary, and the underlying rationale was to balance simplicity, efficiency, and interpretability. Ridge regression is simple and interpretable, and when it works it is our go-to method. A recursive feature machine (RFM) is used for efficiency ‒ our original brute force search [[Einav 2023](https://www.sciencedirect.com/science/article/pii/S2667237523001777)] took ~20x longer and was slightly less accurate. Studies can be weighed and combined in a modular fashion, so that new studies can be added without needing to retrain the model (see comment #6 below). We further note that each component can be changed: For example, a random forest can be used instead of ridge regression, a different feature selection algorithm could be used instead of RFM, or the entire algorithm could be made Bayesian.

Ultimately, an algorithm should be judged for its performance. Across 165,000 HAI titers from 25 datasets, we achieve 2.0x prediction error, comparable to the ~2x accuracy of the HAI assay itself. This provides strong justification that the current algorithm is robust and accurate.

We have elaborated upon these points in the **Methods**:

- Lines 546-548: “CAPYBARA was designed to balance simplicity, efficiency, and interpretability. RFM and ridge regression ensure that the minimum number of predictive features are used, although each component could be modified independently (*e.g.*, using random forests instead of ridge).”
- Lines 552-556: “Predictions between two studies are carried out independently of any other studies. Hence, studies can be weighed and combined in a modular fashion, and introducing a new study does not require rerunning the model on prior datasets. A new study’s predictions for a study-of-interest can be directly combined with all prior predictions using Bayesian inverse-variance weighing (as in Step 4).”

2) I could not get the GitHub notebook to work directly — for example, the torchmetrics package is not specified in requirments.txt so the import statement failed. Even after fixing that, other import errors arose. This was done in a virtual environment with python 3.10 as suggested, so environmental conflicts are not likely to be the issue.

*Author response*: We apologize for this oversight, and we have now fixed the GitHub code and tested it on both Windows and Mac machines. It should now run seamlessly and reproduce all of our analyses and figures.

3) It does not appear that batch correction or “normalization” methods were applied to the data sets from different studies. Can you explain why this is not feasible or necessary or useful?

*Author response*: An excellent question. In the field, HAI titers are considered to be measured on an absolute scale ‒ for example, an HAI titer=40 is correlated with 50% protection. Experimentally, this is checked using controls (*e.g.*, positive control influenza A Reference Goat Antiserum from the International Reagent Resource) to keep values consistent across studies. Achieving accurate predictions on unnormalized data is always preferred (to not discard information), and the fact that our ≈2x prediction accuracy is comparable to the experimental noise of the HAI assay demonstrates that normalization was not required for these 25 studies.

We further note that if a future dataset will have systematic error (*e.g.*, all titers are measured 4x larger than ground truth), our approach would still accurately predict such cases. More precisely, ridge regression is trained internally within each dataset, with the HAI titers of one variant predicted using titers from other variants in that same study. When this ridge model is then applied to another dataset, a global scaling of all titers would automatically be accounted for (aside from a small constant value that typically plays a minor role). As an example, if we take one dataset such as 2010 Fonv_Vac_ and rescale all titers in this dataset by 4x, then predictions from all other studies would still remain nearly as accurate with *σ*_Actual_ minimally changing from 1.9x to 2.0x (**Figure S9A**, *left* shows prediction on original data, *right* on data multiplied by 4x). Repeating this same process in the 2016 Fox_HCW,Vac_ study also leaves the prediction error essentially unchanged (*σ*_Actual_=2.1x to 2.0x, **Figure S9B**). The same holds for all other datasets, showing that this approach is robust.

In addition to **New Figure S9**, we added the following to the **Results** and **Methods**:

- Results, Lines 273-275: “Systematic shifts (*e.g.*, all titers increased by 4x) are automatically accounted for, since HAI titers for one variant are inferred relative to the titers from other variants in that same study (**Fig S9**).”
- Methods, Lines 508-509: “Batch correction across studies was not applied, since HAI titers are standardized using reference antisera to maintain comparability across experiments.”

**Figure S9.** **Multiplying all HAI titers in one study does not significantly change prediction accuracy**. Predicted versus measured HAI titers for two representative vaccine studies using all other studies for training. Titers are predicted in (A) 2010 Fonv_Vac_ and (B) 2016 Fox_HCW,Vac_ using the original data (*left*) or after multiplying all titers in that single study by 4x (*right*) to demonstrate the effects of one study having systematically higher titers.

4) The term “feature” is ubiquitous but not very well-defined. It appears to refer to the HAI titer of a variant, but this is easily missed by the reader. Can you provide a short description of the features used to help the reader?

*Author response*: The reviewer is correct ‒ model features were the HAI titers from influenza variants, and we have now clarified this in the main text. We still opt to keep the term “feature” to emphasize that this approach can be applied in contexts beyond influenza HAI titers.

We have added the following line in the **Results** and modified the **Figure 1 caption** as emphasized below in underlined text:

- Lines 130-132: “Model features are the titers against different influenza variants, with HAI against *V*_1_, *V*_2_, *V*_3_… used to predict the withheld HAI titer against *V*_0_.”

**Figure 1. Combining datasets using CAPYBARA to predict new antibody-virus interactions.** (A) Given studies (...*S_j_*_-1_, *S_j_*, *S_j_*_+1_…) measuring serum HAI against a subset of influenza variants *V*_0_-*V_n_*, and study-of-interest *S*_0_ measuring HAI against *V*_1_-*V_n_*, CAPYBARA predicts *V*_0_’s measurements in *S*_0_. (B) CAPYBARA first identifies the most predictive features (HAI titers against a subset of variants) using Recursive Feature Machines (purple). Ridge regression is applied using those features, training on a subset of data in *S_j_* and cross-validating on the rest (error *σ*_Internal_). This model predicts titer values *μ_j_* from *S_j_* → *S*_0_ without uncertainty. (C) To estimate cross-study prediction error, every other variant is withheld and predicted from *S_j_* → *S*_0_ to determine the internal (*σ*_Internal_) and cross-study (*σ*_External_) error. Combining the errors from every overlapping variant yields the transferability function *f_j_* that is applied to *V*_0_’s *σ*_Internal_ from Panel B to estimate the uncertainty *σ_j_* in *S_j_*. (D) Predictions from all studies are combined through a Bayesian approach to yield a consensus prediction for the study-of-interest (*S*_0_).

5) Since other features like “age” and “year of study” influence prediction accuracy, why not include these as additional features in CAPYBARA?

*Author response*: We appreciated this suggestion and explored it extensively. It turned out that incorporating age information led to *worse* predictions, but in a very interesting way.

We first added each subject’s age along with their HAI titers as potential features that could be selected, but otherwise let CAPYBARA run as in our original approach. During feature selection, age was only picked out in 21%=118/552 of cases (counting each overlapping virus and every pair of studies), and in the cases where it was chosen prediction accuracy either did not change or became *worse* [explained further below] (**Extra Figure 1A vs 1B**, top and middle rows).

We next forced age to be used in all predictions, and found that multiple studies had far worse predictions with error as high as 18.6x, and no predictions became substantially more accurate (**Extra Figure 1A vs 1C**, top and middle rows).

These worse predictions occurred when training on children data (*i.e.*, *Ert* or *Hin* in **Extra Figure 1**) to predict adult data, or conversely using adult data to predict children data. The ultimate explanation was that a regression model fit on children, such as log_2_(HAI/5) = 0.1×age + ··· [HAI features] ···, performed well within the *Ert* study (where children had ages 2-17) but extrapolated to impossibly large HAI titers in adults studies (where ages go as high as 85). While clipping the resulting HAI predictions would minimize the damage, age information always led to worse predictions across age groups, while predictions within age groups (children-children or adult-adult) were minimally affected. We attributed these results to the relatively small effect of age on HAI titers, where titers in the elderly are ≤2x smaller than titers in the young [[Stacey 2025](https://doi.org/10.1016/j.ebiom.2025.105744)].

That said, an incredibly satisfying aspect of this trial was that when combining predictions across all studies, we still achieved ≈2-fold prediction accuracy for each study (**Extra Figure 1**, bottom row). The underlying reason was that although child-to-adult predictions were extremely poor, our CAPYBARA framework inferred that such predictions would be poor (by trying to predict all other variants between those studies using age as a feature), and hence it heavily down-weighed these inaccurate child-to-adult and adult-to-child predictions during the Bayesian weighing. Thus, despite age information inadvertently making many of the predictions worse, CAPYBARA was still robust enough to utilize the best possible predictions in each case.

Lastly, we note that we repeated this process for sex and BMI, and none of these improved prediction accuracy. Year of study could not be directly added because our framework is based on pairwise predictions between two studies, and for any pair of studies this difference is constant. While we could force this information during the Bayesian weighing, the power of our data-driven approach is that it does not have to be biased by our preconceived importance of year-of-study, and as shown in the age example above, it remains robust even when adversarial information is added.

We have added the following to the **Methods** section *Analyzing HAI Titers*:

- Lines 511-519: “We tested if including demographic covariates (age, sex, BMI) as additional features in the ridge regression improved prediction accuracy. Sex and BMI led to comparable prediction accuracy. Age led to far *worse* prediction accuracy when extrapolating from adults→children or children→adults studies, since linear regression is poorly suited to such large extrapolations. However, when combining predictions from all studies, even with age information, CAPYBARA up-weighed the more accurate children→children or adult→adult predictions and retained ≈2-fold prediction error in every study. Although demographic information was removed during the analyses above, these results demonstrate that titers can be robustly predicted even when adversarial information is added, provided that at least some studies yield accurate predictions.”

**Extra Figure 1.** **Effect of including age on cross-study transferability and combined predictions**. Columns show three modeling choices: (A) HAI titers alone as features, as in the main text. (B) HAI titers and age as features, where any feature can be selected using RFM as in the main text. (C) HAI titers and age as features, with age always forced to be a feature. For each modeling choice, the *top row* shows cross-study prediction accuracy *σ*_Actual_ for all pairwise train→test dataset pairs. The *middle row* shows the corresponding pairwise predicted vs. actual HAI values. And the *bottom row* shows the combined predictions obtained by Bayesian weighting across all datasets.

6) Can new vaccine/infection studies with HAI data be easily added to CAPYBARA using Bayesian updating, or must the algorithm be re-trained?

*Author response*: One of the strengths of our approach is that new datasets can be added **without re-training**. Suppose HAI data has been predicted in study *S*_0_ using studies *S*_1_, *S*_2_…*S*_9_, and we are given a new study *S*_10_. After predicting titers from *S*_10_→*S*_0_, CAPYBARA integrates these new predictions with all prior predictions without retaining earlier models. More precisely, if one subject’s titers against one virus were previously predicted to be *μ*_1_±*σ*_1_, *μ*_2_±*σ*_2_, ... *μ*_9_±*σ*_9_, while the new study’s predictions are *μ*_10_±*σ*_10_, the combined predictions would now be

(Σ*_j_*_=1_^10^ *μ_j_*/*σ_j_*^2^)/(Σ*_j_*_=1_^10^ 1/*σ_j_*^2^) ± 1/(Σ*_j_*_=1_^10^ 1/*σ_j_*^2^)^1/2^. [Equation 1]

We have clarified this point in the **Methods** section:

- Lines 550-554: “Predictions between two studies are carried out independently of any other studies. Hence, studies can be weighed and combined in a modular fashion, and introducing a new study does not require rerunning the model on prior datasets. A new study’s predictions for a study-of-interest can be directly combined with all prior predictions using Bayesian inverse-variance weighing (as in Step 4).”

7) Can you provide a clearer explanation of what CAPYBARA revealed about the cross-reactivity landscape? For example, does it provide useful guidance for designing the next seasonal or universal influenza vaccine?

*Author response*: This was a great point, and it synergized with Reviewer #3’s Comment #1. Briefly, we determine which subset of amino acid positions on influenza hemagglutinin have sequence edit distance that best approximates the CAPYBARA-derived virus-virus importance relations, identifying the key residues that underlie the cross-reactivity relations. We invite the Reviewer to read our full response below in Reviewer #3’s Comment #1.

Reviewer #2

This paper presents a method for out of sample prediction of serological data, with the ability to combine multiple datasets and preferentially weight studies and factors with more predictive information. This is an important problem in the field as it is highly challenging to reach very large sample sizes in a single human cohort due to practical limitations, so developing methods to incorporate data from and work across multiple cohorts is highly valuable in computational immunology. The code for this work is publicly available and well documented, and the manuscript describes the approach somewhat clearly and tests multiple use cases. I have outlined my comments below, but overall I found this to be a nice manuscript.

Major Comments

1) I feel it would be very helpful to include a table/graphic/figure explaining the precise definitions of *σ*_internal_, *σ*_external_, *σ*_predicted_, and *σ*_actual_. They are defined in the text, but I think this would help with readability. Especially as the relatively low error of the predictions is central to the story of the paper.

*Author response*: This was a solid point, and we added a **new Table 1** defining each term:

- Lines 147-148: “**Table 1** describes the four *σ* terms used throughout this study.”

**Table 2.** **Definitions of CAPYBARA error terms and their roles in model training, transferability, and evaluation.** Models are trained to infer HAI titers for variant *V*_0_ in study *S_j_* and then applied to predict *V*_0_‘s titers in study *S*_0_. Titers from other variants *V_k_* can be chosen as model features.

| **Symbol*** | **Description** | **How it is computed** | **Where it is used** |
| --- | --- | --- | --- |
| *σ*_Internal_ | *Within-study* prediction error in *S_j_* | RMSE on held-out subjects during internal cross-validation (train 80%, test 20%) | *x*-axis in transferability function to compute *σ*_Predicted_ from *S_j_→S*_0_ |
| *σ*_External_ | *Cross-study* prediction error in *S*_0_ | By predicting every other virus *V_k_* in *S*_0_ (while withholding *V*_0_) | *y*-axis in transferability function to compute *σ*_Predicted_ from *S_j_→S*_0_ |
| *σ*_Predicted_ | Estimated prediction error in *S*_0_ | Using *σ*_Internal_ and *σ*_External_ from all other *V_k_*’s, and *σ*_Internal_ from *V*_0_ | Compute for each *S_j_*, then combine using Bayesian weighing (weight ∝ 1/error^2^) |
| *σ*_Actual_ | Ground truth prediction error in *S*_0_ | RMSE between predicted and measured titers for *V*_0_ | Determine prediction accuracy |

*All RMSEs are computed on log_2_(HAI/5) and then unlogged through exponentiation by 2.

2) It is a known issue with HAI assays that variability in the assay can come from many factors including site or technician. I wonder if the error estimates generated by this method could be used to develop weights or adjustments between cohorts that could allow them to be combined for other analyses. It would be interesting to see some sort of systematic look at the magnitude and sign of the residuals for particular prediction tasks.

*Author response*: This is an interesting suggestion. If there was systematic error between two datasets (*e.g.*, one study has all titers increased by 4x), then our method would account for this and still yield the same prediction accuracy (see Reviewer #1’s Comment #3). Thus, when predictions between two studies are poor, it means the cross-reactivity relations in those two studies differ (as a simple example: HAI_Virus 1_=HAI_Virus 2_+HAI_Virus 3_ in one study but not another). This would make it difficult to combine such data. However, we can quantify when studies have different cross-reactivity relations, and we do so below in two ways.

First, for each pair of studies, we computed the mean signed prediction error for each train→test dataset pair as log_2_(measured HAI/predicted HAI) (see the **New Figure S4** panel below). Notably, the *All Datasets* row at the top (showing combined predictions trained on all other studies) is always close to zero, implying little-to-no systematic bias. However, there were a few rows ‒ most notably 2012 Ert_LAIV,Vac_ and 2013 Ert_LAIV,Vac_ ‒ that showed consistent deviations.

**Figure S4.** **Prediction accuracy between every pair of studies.** Signed prediction error on log titers, log_2_(measured HAI/5)-log_2_(predicted HAI/5) for all variants in each pair of studies. Red indicates that measured titers were larger than predicted titers on average. Training is either done using all studies (top row) or using a single study (all other rows).

As expected, the relationships between the HAI titers of influenza variants were very different in these two Ert_LAIV_ studies. More precisely, titers were nearly flat for all variants between 1968-2002, often hitting the HAI=5 limit of detection, and then rose slightly for variants from 2004-2014 (**Extra Figure 2A,B**). In contrast, adult data from this same manuscript (and from all other studies) had titers above the limit of detection for most variants (**Extra Figure 2C,D**). Thus, it makes sense that HAI titer relationships would markedly differ between these two types of studies, since the former is hitting the limit of detection. Biologically, the small HAI titers in the two Ert_LAIV_ studies make sense, since children are not only expected to have low HAI titers for older variants circulating before they were born, but LAIV generally elicits little-to-no titers in adults or children that have received prior influenza vaccinations, and even in children receiving their first vaccine only ~33% of them show HAI titers [[Hao 2025](https://www.jci.org/articles/view/189300)], all of which would lead to low HAI titers.

**Extra Figure 2.** **HAI titers from the four Ert vaccine studies.** Children data from (A) 2012 and (B) 2013 receiving the live-attenuated influenza vaccine (LAIV) exhibit low HAI titers against most viruses. Adult data from (C) 2010 and (D) 2013 receiving an inactivated influenza vaccine (IIV) have uniformly higher titers. In all panels, gray denotes the pre-vaccination titers while the colored lines denote post-vaccination titers. Figure adapted from [[Ertesvag 2022](https://pubmed.ncbi.nlm.nih.gov/35750781/)].

As a second complementary analysis, we visualized the mean Bayesian weights (1/*σ*^2^) for each dataset pair across all overlapping viruses, representing how much each study contributes to predictions in the other studies (see **Figure S4A** below). There was always at least one training dataset for each test dataset with 1/*σ*^2^≥0.9, and these highly-predictive datasets tended to be collected within 10 years of the test dataset. Beyond that trend, we did not find any patterns in which datasets were more predictive. Indeed, there were multiple examples where one dataset was highly predictive for a group of studies (*e.g.*, 2018 UGA_Vac_ predicts both Hay and all five Ert studies exceptionally well), while neighboring studies that would be expected to be identical (*e.g.*, 2016 UGA_Vac_ and 2017 UGA_Vac_) showed poor predictive power against those same studies. To summarize these results: 1) a main strength of CAPYBARA is that it can unbiasedly detect such differences between datasets and use the titers from the most predictive studies, 2) the most predictive studies are often conducted within 10 years of each other, although not all studies conducted ≤10 years apart are highly predictive. We hope that as our compendium of datasets continues to grow, we will find additional rules that can help explain when two studies will predict one another, and we have added that statement to the **Discussion**.

We have modified the **Results** and **Discussion** as follows:

- Results, Lines 234-238: “The greatest signed error deviations occurred when the two 2012-2013 Ert_LAIV,Vac_ studies were used for training where, unlike in all adult studies, HAI titers for nearly all viruses hit the limit of detection (HAI=5), leading to markedly different cross-reactivity relations. Even so, the combined predictions using all studies uniformly had signed error ≈0, demonstrating improved accuracy when using more datasets (**Fig S4**).”
- Results, Lines 240-246: “Measured accuracy dropped, often significantly, when using older datasets to predict more recent ones. In particular, training the oldest 1996–2000 datasets led to poor predictions and large variation on 2011–2015 (*σ*_Actual_ = 3.2x; *p*<0.05, two-sided permutation test) or 2016–2020 data (*σ*_Actual_ = 2.6x; *p*<0.05, two-sided permutation test), although predictions going backwards in time by >10 years tended to be more accurate. Estimated accuracy (1/*σ*_Predicted_^2^) showed similar behavior, with larger confidence for studies conducted within 10 years of one another (**Fig S4**).”
- Discussion, Line 446-448: “However, studies conducted within 10 years were not always highly predictive, and future studies should explore what other study features lead to similar cross-reactivity relations and better predictive power.”

**Figure S4.** **Prediction accuracy between every pair of studies.** (A) The estimated prediction accuracy for each pair of studies, computed as the mean 1/*σ*_Predicted_^2^ over all overlapping variants. Larger values indicate that the training dataset will be weighted more heavily in combined-study predictions. (B) Signed prediction error on log titers, log_2_(measured HAI/predicted HAI) for all variants in each pair of studies. Red indicates that measured titers were larger than predicted titers on average. Training is either done using all studies (top row) or using a single study (all other rows).

3) Tying into my above comment about understanding these errors, by eye it seems the approach has the largest challenges with the highest and lowest titer values. However it may be the case that it is an optical illusion due to all of the errors being in the same direction. Of course, necessarily, error in predicting undetectable titers must be an overestimate. I would like to see a figure characterizing the error by titer value. It’s not an issue in my eye if this is in fact the case, we know HAI limits of detection make these problems challenging, but it would be good to show.

*Author response*: This was not an optical illusion. For measured HAI titers between 10 and 320 (which represents 74% of all titers), the measurements fell between the lower and upper quartiles of all predictions, but for smaller and larger measurements the predictions became skewed (see **New Figure S3**). As the reviewer pointed out, we often see this behavior at the edge cases of predictions, especially when values are censored (*i.e.*, any HAI titer below the limit of detection is assigned HAI=5).

This is a good caveat to point out, and we added the **New Figure S3** and modified the **Results**:

- Lines 228-232: “Computing signed prediction error showed that predictions were skewed to be slightly larger when the measured titer=5 and slightly smaller when the measured titer was for higher ≤640, yet the median predictions were within 2-fold for measured titers ≤1280 and within 4-fold for larger titers, and the mean signed error was ≈0 across most studies (**Fig S3, Fig S4**)**.”**

**Figure S3.** **Signed prediction error as a function of the measured ground truth titer**. Boxplots show median and interquartile range of prediction residual across all datasets, with sample counts annotated above each bin.

4) Is it necessary with this method to impute the missing HAI measurements and predict them/use them for prediction? If it is necessary to do the ridge regressions or feature selection that is okay, but it feels important to acknowledge this.

*Author response*: We thank the reviewer for raising this important point. Yes, imputation is required for CAPYBARA because both the Recursive Feature Machine (RFM) and ridge regression require complete data. However, these imputed values are ignored when evaluating model accuracy, and only measured values are used to compute all of the *σ* variables described in Comment #1 above.

In terms of the imputation method, our approach used row- and column-wise mean, and the resulting prediction accuracy (prediction error≈2x, comparable to the intrinsic error of the HAI assay) demonstrates that this type of imputation worked well. For completeness, we also verified that an alternative *k*-nearest-neighbor imputation approach yields comparable results.

We have clarified the **Results** and **Methods** as follows:

- Results, Lines 145-147: “Missing HAI entries were imputed using the row- and column-means of all measured values, yet all error metrics (*e.g.*, σ_Actual_) were computed using the measured titers.”
- Methods, Lines 496-5499: “Missing HAI titers, comprising 2.1% of all measurements, were imputed using the row–column mean since both RFM and ridge regression require complete data. While these imputed values were used for model training, model evaluation was only carried out on the measured values.”

Minor Comments

1) Extra word: ”This determines how the error relationship when predicting from *S_j_* to *S*_0_ (Fig 1C)...”

*Author response*: Thank you, this has been fixed.

2) “Prediction accuracy can only decrease as more datasets are included”. Increased?

*Author response*: This has also been fixed.

Reviewer #3

The manuscript by Orsinelli-Rivers describes the development of an analytical workflow for modeling and mapping the relationships among different influenza virus serological datasets. I commend the authors for tackling this important problem and for providing an initial framework to infer influenza virus cross-reactivity from existing data. However, an important issue not addressed in the study is the accuracy of the model’s predictive power in relation to antigenic changes. Notably, one of the main conclusions is that predictions are accurate when datasets are derived from studies conducted less than 5 years apart. This inherently suggests that serological assays performed on antigenically similar viruses, typically those within a 5-year window, are more likely to yield more accurate cross-reactivity predictions. Another important point not addressed in the study is the generalizability of this analytical workflow, despite this being emphasized in both the abstract and the title of the manuscript. These and other issues included below, should be addressed before the manuscript can be considered for further evaluation.

Major comments:

1) While the analytical workflow is useful for predicting serological cross-reactivity among H3N2 influenza viruses, it has a major limitation regarding the biological interpretation of antigenic differences between the viruses assessed. As shown in Figure 4, the accuracy of predictions was influenced by the time elapsed between the datasets used for analysis and the variants being evaluated—viruses within a five-year window were more likely to yield accurate cross-reactivity predictions. This likely reflects lower antigenic divergence within such a timeframe; in other words, H3 hemagglutinins (HAs) within five years of each other are generally more antigenically similar, with this similarity declining beyond that period. This trend is clearly visible in Figure 5 and Supplementary Figure S5. However, there are notable exceptions—some viruses that are many years apart still produce accurate predictions. This discrepancy is not addressed in the study. It would be helpful for the authors to explore this further, possibly by analyzing HA sequences to identify amino acid residues that may underlie cross-reactivity between antigenically distant viruses. An amino acid sequence alignment could provide insights into this issue. While it is understandable that the primary focus of the manuscript is the development of the analytical workflow, the biological interpretation of the results should not be overlooked. Including such analysis in future iterations of the tool could improve its utility and generalizability. This is particularly important given the history of antigenic shifts in influenza viruses—for example, the 2009 H1N1 pandemic virus exhibited close antigenic similarity to strains that circulated prior to the 1968 H3N2 pandemic, highlighting the complexity of antigenic evolution across time.

*Author response*: We thank the reviewer for this insightful comment, and we performed two complementary analyses to identify which HA amino acid positions recoup the cross-reactivity relationships revealed by our approach. Since the HAI assay we use only measures antibodies targeting the hemagglutinin head (HA1), we focus exclusively on hemagglutinin positions 68 to 292 comprising this part of the protein.

Method #1: *Minimal Key Residues*

We first identified the minimal set of HA1 residues whose edit distance was as similar as possible to the RFM-derived virus-virus similarity matrix. In other words, in cases where two viruses accurately predict one another’s HAI titers, those viruses should have similar sequences at the sites targeted by antibodies. Hence, we used backwards-elimination to identify these sites, starting from the full HA1 sequence and removing one amino acid at a time that led to the best improvement between the edit‒distance-derived virus similarity matrix and the RFM similarity matrix using the Frobenius norm.

More precisely, for two influenza variants *V_j_* and *V_k_*, we compute their mean RFM importance (*importance_jk_*) across all studies. Using the HA1 amino acids at sites {*s*_1_, *s*_2_, *s*_3_…} (where we start with full HA1 and drop one amino acid at a time), we align and compute their edit distance at these sites (*edit_jk_*). We then compute the difference between the matrices

|*importance* - *edit*|_Frobenius_ = [∑*_jk_* (*importance_jk_ - edit_jk_*)^2^]^1/2^*.* [Equation 2]

**Extra Figure 3** shows the resulting Frobenius norm as each additional site is removed.

**Extra Figure 3. Identification of HA1 head positions that best reproduce RFM-derived virus-virus similarity.** Removal of one amino acid’s position at a time from the full HA1 head sequence that led to increased similarity (lowest Frobenius norm) between RFM importance and sequence-based edit distance. The dashed line marks the final set of key amino acids, past which the Frobenius norm starts to increase.

The minimum Frobenius norm was achieved with a concise subset of 31 amino acid sites that produced edit distance similarity (**New Fig S11A**, shown below) roughly comparable to RFM-derived similarity (**Fig 5A**, also shown below for reference). As expected, many variants circulating within the same 5 years had similar sequences and thus large similarity, but because we subset to key residues, we also saw some far-range interactions. This method represents the smallest set of amino acids that mediates antibody cross-reactivity.

Method #2: *Conserved Sites in Distant Variants*

Whereas method #1 considered all pairs of viruses (including those circulating a few years apart), we also did a complementary analysis specifically looking at pairs of variants circulating 10+ years apart that were highly predictive of one another. We took all 11 such pairs of variants with high predictive accuracy (RFM importance ≥0.7), aligned their HA1 sequences, and noted which HA1 sites were found in over 80% of such pairs, identifying a total of 167 sites. This number was far larger because there were only 11 pairs, and hence these variants explore a limited part of sequence space. As expected, maximum edit distance across all 167 sites was at most 6 for any pair of variants, since we are enriching for highly conserved sites among viruses circulating many years apart (**Fig S11B**).

Since Method #1 main was skewed towards the more numerous pairs of viruses circulating a few years apart, while Method #2 focused on viruses circulating many years apart, we took the intersection of both sets to find 21 sites that both methods suggested are key targets for the antibody response (**Fig S11C,D**). As a further check, 14 residues fell within the canonical H3N2 epitopes A-E and the receptor binding site, highlighting their biological plausibility. 3 additional sites lie outside these canonical sites, but have been previously reported to impact H3N2 antigenicity [[Wolf 2006](https://biologydirect.biomedcentral.com/articles/10.1186/1745-6150-1-34), [Shih 2007](https://www.pnas.org/doi/full/10.1073/pnas.0701396104), [Ndifon 2009](https://www.pnas.org/doi/full/10.1073/pnas.0903427106), [Koel 2013](https://www.science.org/doi/10.1126/science.1244730), [Fox 2022](https://www.mdpi.com/1999-4915/14/3/470)]. We also identified 3 sites outside of the canonical epitopes that we have not seen previously reported. Taken together, the HAI data suggest that these 21 sites are responsible for both the short-range and long-range similarity between influenza variants.

We have added a **New Figure S11** and modified the **Results**, **Discussion,** and **Methods**:

- Results, Lines 374-382: “Finally, we determined the key HA sites that underlie influenza cross-reactivity in two different ways. Method #1 identified the smallest number of HA1 amino acids whose edit distance best matches the RFM-derived virus-virus similarity (a.k.a. *Minimal Key Residues*). Starting from the full HA1, we eliminated one amino acid at a time that led to the greatest similarity (smallest Frobenius norm) between virus edit distance at the remaining sites and RFM importance. This identified a small set of 31 HA1 positions, where the virus-virus edit distance at these sites (**Fig S11A**) roughly reproduced their RFM-derived feature importance (**Fig 5A**) with Pearson correlation *r*=0.60. In both methods, we found the expected similarity between contemporaneous variants as well as between some variants circulating 10+ years apart.”
- Results, Lines 384-390: “Method #2 identified the key conserved residues across distant variants necessary for accurate predictions (a.k.a. *Conserved Sites in Distant Variants*). Thus, we analyzed the 11 pairs of variants circulating 10+ years apart with ≥0.7 RFM importance. Taking the most common conserved sites in >80% of pairs identified 167 amino acids, with this larger number arising because the 11 pairs explored a limited part of sequence space. Across all variants, the edit distance at these sites was at most 6, demonstrating that these are highly conserved sites in HA1 (**Fig S11B**).”
- Results, Lines 392-399: “When we compared the residues found from each method, we found that 21 of them overlapped, representing the positions most likely to be important for both variants circulating in similar years as well as 10+ years apart (**Fig S11C,D**). As a further check, 14 residues fell within the canonical H3N2 epitopes A-E and the receptor binding site, highlighting their biological plausibility. 3 other sites lie outside these canonical sites but have been previously reported to impact H3N2 antigenicity, while 3 additional sites outside the canonical epitopes were identified by this analysis [[Wolf 2006](https://biologydirect.biomedcentral.com/articles/10.1186/1745-6150-1-34), [Shih 2007](https://www.pnas.org/doi/full/10.1073/pnas.0701396104), [Ndifon 2009](https://www.pnas.org/doi/full/10.1073/pnas.0903427106), [Koel 2013](https://www.science.org/doi/10.1126/science.1244730), [Fox 2022](https://www.mdpi.com/1999-4915/14/3/470)]. Taken together, the HAI data suggest that these 21 sites are responsible for both the short-range and long-range similarity between influenza variants.”
- Discussion*,* Line 437-439: “The resulting cross-reactivity relations could be partially recouped by a subset of 21 amino acids in the HA head, offering a direct link between model-derived relationships and underlying sequence features.”

**Figure S11. Identification of HA1 head positions that best reproduce RFM-derived virus-virus similarity.** Arc plots showing edit distance in the subset of key HA1 sites identified using (A) *minimal key residues* [31 sites, lowest Frobenius norm between sequence distance and RFM importance] or (B) *conserved sites in distant variants* [167 sites, variants 10+ years apart with high importance]. (C) Residues identified by both methods shown on a representative sequence for H3N2 A/Perth/16/2009, with the canonical epitopes denoted by different colored lines above each position. Three other residues identified in prior studies (gray) or this study (pink) are also shown. (D) 21 overlapping HA1 amino acids from both methods, annotated with the H3N2 epitope they fall into. Amino acid positions all use H3N2 numbering.

**Figure 5. A global dictionary of influenza variant importance.** (A) Rainbow diagram of feature importance between any pair of variants (connections are bidirectional).

2) While the authors mention that this analytical tool is generalizable, they did not provide evidence that the workflow works effectively for other influenza subtypes (*e.g.*, H1N1) or other viruses. Therefore, it would be more appropriate to avoid making this assumption in the manuscript. I would also recommend a slight revision of the title to better reflect the specific scope and finding of the study: “CAPYBARA: A Framework for Predicting H3N2 Influenza Serological Measurements Across Human Cohorts”.

*Author response*: We agree that the original manuscript only considered H3N2 data, and we took this opportunity to extend our analysis to include HAI titers for influenza H1N1, B Victoria, and B Yamagata from the datasets we analyzed (see **New Figure S7** below). The model achieved comparable predictive accuracy across all additional viruses, with RMSE between measured and predicted values between 2-3x, similar to what we observed for H3N2. These results make a very strong case for the general applicability of CAPYBARA to influenza.

Accordingly, we have retained the original title and have revised the **Results** and **Methods** to clarify that the framework has been empirically validated for multiple influenza subtypes and is in principle extensible to other viral systems:

- Results, Lines 262-267: “To demonstrate the generalizability of this approach, we next used CAPYBARA to predict all H1N1, B Victoria, and B Yamagata HAI titers in these same studies. Although the virus panels were smaller in each case, the UGA studies had the necessary overlap of ≥3 variants. Combined predictions of HAI titers using all datasets led to *σ*_Actual_=1.9-2.6x for H1N1, comparable to H3N2 prediction accuracy (**Fig S7**). B Victoria and B Yamagata achieved *σ*_Actual_=1.7-3.7x, with most studies against being predicted with ≈2-fold error (**Fig S7**).”
- Methods, Lines 487-490: “To assess subtype generalizability, CAPYBARA was applied without modification to HAI titers for H1N1, B Victoria, and B Yamagata in these same datasets. Each subtype or lineage was predicted independently, with model fitting, cross-dataset prediction, and error estimation performed identically to the H3N2 analysis.”

**Figure S7.** **Predicting HAI responses across other influenza subtypes**. (A) Heatmap of the predicted vs measured RMSE (*σ*_Actual_) across all subjects and overlapping variants for H1N1 (left column), B Victoria (middle column), and B Yamagata (right column). Within each heatmap, training is either done using all studies (top row) or using a single study (all other rows). (B-C) All predicted vs measured HAIs when training on (B) a single study or (C) all other studies. The number *N* of predictions is larger for pairwise predictions since the same serum-virus pair is predicted multiple times using different training datasets. The diagonal line *y*=*x* represents perfect predictions.

Minor Comments:
1) In the introduction, the authors should indicate that the study was performed only with H3N2 viruses.

*Author response*: As addressed in Major Comment #2 above, our analysis now includes H1N1, B Victoria, and B Yamagata data. The **Introduction** has been revised accordingly to reflect this broader scope:

- Lines 87-88: “We first apply CAPYBARA to H3N2 data and then demonstrate its generalizability by predicting H1N1, B Victoria, and B Yamagata titers.”

2) The second paragraph of the introduction indicates “While thousands of new variants (or strains) emerge each year, …”. Please provide a reference for this statement.

*Author response*: We appreciate this comment and have added an appropriate reference to the **Introduction** to support this statement. Specifically, we point to H1N1 [[Harvey 2016](https://journals.plos.org/plospathogens/article?id=10.1371/journal.ppat.1005526)] and H3N2 data [[Harvey 2023](https://journals.plos.org/ploscompbiol/article?id=10.1371/journal.pcbi.1010885)] from the Crick Center, one of the WHO centers for influenza surveillance that assesses thousands of emerging influenza variants each year, as well as Next Strain [[Lee 2023](https://www.frontiersin.org/journals/bioinformatics/articles/10.3389/fbinf.2023.1069487/full)] that provides one of the best-in-class visualization tools of these influenza variants:

- Line 59-61: “While thousands of new variants (or strains) emerge each year [[Harvey 2016](https://journals.plos.org/plospathogens/article?id=10.1371/journal.ppat.1005526), [Harvey 2023](https://journals.plos.org/ploscompbiol/article?id=10.1371/journal.pcbi.1010885), [Lee 2023](https://www.frontiersin.org/journals/bioinformatics/articles/10.3389/fbinf.2023.1069487/full)], only a small fraction can be functionally characterized using HAI, and the variants measured often differ between studies.”

3) The fifth paragraph from the introduction, which starts with: “A key innovation…” provides discussion points of the workflow, and therefore this should be moved to the discussion section.

*Author response*: We appreciate and agree with this comment, and have split this paragraph across the Results and Discussion sections, as appropriate.

4) I am a bit confused by the last sentence of the introduction, as the authors did not specifically quantify differences among study populations, experimental conditions, and virus panels. Did they mean to say that, despite differences among study populations, experimental conditions, and virus panels, the analyses workflow allows to estimate cross-reactivity among different Influenza virus serological datasets? I suggest revising this sentence. Also edit the beginning of the sentence to read: “By predicting these interactions for H3N2 viruses, …”

*Author response*: The Reviewer is correct, and we have revised this sentence as follows:

- Lines 104-106: “Accurate cross-study predictions in the face of differences in study populations, experimental conditions, and virus panels would not only expedite future experiments but also help quantify the magnitude and breadth of the immune response in greater resolution.”

5) In the second paragraph, line 2 of the discussion, did the authors mean to say “…cross-reactivity across influenza variants.”?

*Author response*: Good catch. The Reviewer is correct, and this error has been fixed.

6) In the fourth paragraph of the discussion, did the authors mean to say “…didn’t require”?

*Author response*: This has also been fixed.

Reviewer #4

I find this work interesting and I think the manuscript is for the most part well-written.

1.1) There is a sentence, page 8 of the manuscript, "Prediction accuracy can only decrease..." Please clarify this statement, it is vague saying adding more datasets when the number of datasets is not restricted. It lists studies {S1, S2,...} Perhaps this is intentionally left vague because we cannot easily say an exact number of datasets to stop at, however the statement I believe can be made more clear.

*Author response*: These are good points, as there was both a content error and a clarity issue with this paragraph.

First, the sentence should have read “Prediction accuracy can only **increase** as more datasets are included,” and we should have noted that the number of other studies is always restricted to be finite. If we are predicting the HAI titer in study *S*_0_ from *n* other studies, and the prediction from study *S_j_* is *μ_j_*±*σ_j_*, then the final prediction from all studies combined will be

(Σ*_j_*_=1_*^n^ μ_j_*/*σ_j_*^2^)/(Σ*_j_*_=1_*^n^* 1/*σ_j_*^2^) ± 1/(Σ*_j_*_=1_*^n^* 1/*σ_j_*^2^)^1/2^. [Equation 3]

When we add an additional study *S_n_*_+1_, whose prediction must have uncertainty 0<*σ_n_*_+1_<∞, the new error term in Equation 3 must decrease, which implies increased prediction accuracy.

In addition, the Reviewer pointed out that the number of datasets needed was vague. This was an intentional choice, because we cannot know the desired prediction accuracy needed in future applications, nor how good the predictions will be from studies *S*_1_, *S*_2_…*S_n_*. For example, if we desire 2-fold prediction accuracy, but every *S_j_* has *σ_j_*=6x (representing very poor predictions), then we would need *n*=9 datasets to achieve 2x error in Equation 3. On the other hand, if we estimate that the error from *S*_1_ is 2x, then we only need that single (*n*=1) study. We envision that our framework will be used iteratively until the desired prediction accuracy is achieved.

We have modified the **Results** as follows:

- Line 134: Clarifies that the number of studies is restricted. “In every other study *S_j_*∊{*S*_1_, *S*_2_…*S_n_*}”
- Line 141: Fix the content error. “Prediction accuracy should only **increase** as more datasets are included…”
- Lines 272-273: Explain the iterative use. “Thus, more datasets can be added until the desired prediction accuracy is achieved.”

1.2) Later, in the paper there is again mention of very noisy datasets having little impact and it also states all datasets can be included, which seems to go against the statement where adding more datasets will decrease accuracy. Hence, some clarification of these statements would be beneficial to the reader.

*Author response*: As mentioned in our prior answer, we have now corrected the line to explain that adding datasets can only improve prediction accuracy.

In the special case where a new dataset *S_n_*_+1_ is extremely noisy, and hence its estimated error will be large (*σ_n_*_+1_→∞), Equation 3 shows that the combined error from all studies will negligibly change. In other words, adding a very noisy dataset will not hurt the combined predictions. In practice, the error from any study will be finite (6<*σ_n_*_+1_<10 represents very large error), and the overall error in Equation 3 will almost negligibly decrease by adding this dataset.

As a concrete example, if we combine a study with good estimated prediction accuracy (*σ*_1,Predict_ = 2.3x) and a study with poor estimated accuracy (*σ*_2,Predict_ = 7.7x), the combined predictions will show a very small improvement over the accurate study (*σ*_1+2,Predict_ = 2.1x, **Fig S8A**).

Taking an even more extreme example, we can then add a noisy study (*i.e.*, random HAI titers with the same distribution as an actual dataset). Predictions based solely on this noisy dataset are inaccurate (*σ*_3,Predict_ = 7.8x), as expected (**Fig S8B**). However, predictions from all three studies will again show a very small improvement over the accurate study (*σ*_1+2+3,Predict_ = 1.9x, **Fig S8B**). Thus, adding noisy studies did not hurt the estimated prediction accuracy.

Finally, we note that the measured vs predicted HAI titers always followed *σ*_Actual_≲*σ*_Predict_ in these examples, as expected. As a reminder, *σ*_Predict_ was constructed to represent an upper bound or “worst case scenario” error, which holds in each of these examples.

We have updated the **Results** and **Methods** sections as follows:

- Results, Lines 269-272: “Lastly, we showed that this CAPYBARA can disregard an extremely noisy dataset by generating a new study with random H3N2 HAI titers drawn from the same distribution and with the same variants as the 2016 UGA_Vac_ and 2007–2011 Fonv_Inf_ studies (**Fig S8**). Adding this noisy dataset to the existing studies did not affect overall prediction accuracy, as expected.”
- Methods, Lines 521-523: “A synthetic noisy dataset was created by randomly sampling HAI titers from the joint distribution of real training studies (2016 UGA_Vac_ and 2007–2011 Fonv_Inf_), preserving the same virus panel and assuming the average number of subjects between the two studies (214 subjects).“

**Figure S8.** **Predictions remain robust with the inclusion of a noisy dataset.** (A) Example predictions from Fig 2A using an individual dataset (left and middle columns) or the combination of both datasets (right column) to predict titers in 2017 UGA_Vac_. Labels above each plot identify the training → testing dataset. (B) A study with random data predicted this same testing dataset either individually (left column) or in addition with the other two training datasets from Panel A (right column).

2) On page 16 of the manuscript, "The algorithm did require..." It seems you meant to write didn't or did not.

*Author response*: Thank you, this has been fixed.

3) I would be interested in seeing this methodology utilized in other studies for vaccine development. I think with some minor writing revisions this is an acceptable paper.

*Author response*: This is exactly what we plan to do next, and we have elaborated upon this idea in the **Discussion**. More precisely, annual influenza surveillance is carried out by several WHO Collaborating Centers, where each center typically measures human sera against multiple variants of interest to that region (*i.e.*, the Europe center uses viruses predominantly circulating in Europe, the US center uses viruses from the US…). While there are often 1-2 overlapping viruses in these panels, all of the other influenza variants tend to be local to that country; this is an oversight, since in our global world the dominant strain in Europe could become the dominant strain worldwide.

Our approach could help close this gap by using the overlapping variants to predict how each variant tested at one WHO site would be inhibited by the sera at all sites, thereby showing whether the viruses circulating in Europe not only escape antibody immunity in Europeans, but from all major populations around the world.

We have modified the **Discussion** as follows:

- Line 470-477: “In principle, CAPYBARA could help augment vaccine strain selection by unifying the insights gained from influenza surveillance from around the world. For example, each year the WHO Collaborating Centers measure how human sera from their country inhibit potential vaccine strains from their region, resulting in partially overlapping virus panels that are local to each site. If each site agrees to measure the same three viruses (*e.g.*, the most recent three vaccine strains), they could immediately infer how sera measured at any WHO site would inhibit each variant measured at every other site, thereby quantifying the potential for viral escape across the world.”
